# 20-year individual physical activity patterns and related characteristics

**DOI:** 10.1186/s12889-022-12862-1

**Published:** 2022-03-04

**Authors:** Anne Loyen, G C Wanda Wendel-Vos, Maryam Ismaili Shekoh, W M Monique Verschuren, H Susan J Picavet

**Affiliations:** 1 Centre for Nutrition, Prevention and Health Services, National Institute for Public Health and the Environment, Bilthoven, The Netherlands; 2 Institute of Health Sciences, VU University, Amsterdam, The Netherlands

**Keywords:** General population, Lifestyle, Physical activity, Prospective study, Time trends

## Abstract

**Background:**

This study aims to describe individual leisure-time physical activity patterns among Dutch adults over a 20-year period, and to compare baseline characteristics of participants with different patterns.

**Methods:**

The study population consisted of 2,518 adults (53% women) aged 26–65 years at baseline, measured every 5 years over a 20-year period. Self-reported physical activity measurements (from 1994 to 2017) were used to compose five (predefined) patterns: stable active, becoming active, becoming inactive, stable inactive, and varying physical activity. Multivariate logistic regression analyses were used to compare baseline socio-demographic, lifestyle, and health-related characteristics of these patterns.

**Results:**

The total population shows a stable percentage being active in each round (between 55 and 58%). However over a period of 20 years, 32.6% of the participants were stable active, 19.9% were stable inactive, 15.2% became active, 11.6% became inactive, and 20.8% had varying physical activity behaviour. Compared to participants who were stable active, becoming active was associated with being 46–55 years old, having an intermediate level of education, and smoking, at baseline. Participants who became inactive were less likely to be 46–55 years old and more likely to be obese. Stable inactivity was associated with an intermediate level of education, low adherence to dietary guidelines, smoking, low levels of alcohol use and a moderate/poor perceived health. Participants with a varying physical activity level were more likely to have low adherence to dietary guidelines and to smoke.

**Conclusions:**

Almost half of the participants changed their physical activity behaviour over 20 years. Baseline age, level of education, smoking, alcohol consumption, adherence to dietary guidelines, weight status and perceived health were associated with different physical activity patterns.

## Background

Physical activity is an important health-related behaviour. According to the World Health Organization “global recommendations on physical activity for health” adults should accumulate at least 150 min of moderate-to-vigorous physical activity per week [1]. Not meeting these physical activity recommendations, also known as physical inactivity, has been associated with an increased risk of several health conditions, such as cardiovascular disease, type 2 diabetes, certain types of cancer, and mortality [2, 3]. In 2020 the recommendations has been updated [4] though most available studies use the ‘old’ recommendations. Studies have estimated that approximately 30% of the global adult population is physically inactive [5, 6]. Worldwide, physical inactivity is considered the fourth leading risk factor for non-communicable diseases, [2] and is considered to cause millions of preventable deaths [2, 3]. The economic burden of physical inactivity has been estimated to be at least 67.5 billion international dollars [7].

In the Netherlands, 50% of the adults aged 18–64 years adhered to the physical activity guidelines in 2018. For older adults (65 + years), this was 37% [8]. Over the last two decades, both groups showed a (slight) increase in levels of physical activity [8]. These population levels might, however, not reflect individual changes in physical activity over the life course [9].

In 2010, Picavet and colleagues reported on 10-year changes in leisure-time physical activity habits in Dutch adults, using the Doetinchem Cohort Study, and reported that almost half of the population changed from active to inactive or vice versa, indicating that physical activity is a dynamic behaviour. In addition, they reported that not smoking and having a high socio-economic status were associated with staying active, that inactive men were most likely to stay inactive, and that a good perceived health was associated with becoming active [9]. Since 2010, several studies on individual changes in physical activity based on longitudinal data has been published, most of them using advanced statistical methods that calculate a limited number of groups with similar physical activity patterns, often referred to as distinct trajectories. These were summarized in a systematic review by Lounassalo et al. in 2019 (Lounassalo 2019). The PA trajectories found depend highly of the population studied, but always included (some variations of) being stable physical active, stable non active, decreasing activity, increasing activity or a varying pattern. These were also the predefined patterns in Picavet et al. [9]. The ongoing Doetinchem Cohort Study now includes another two waves of measurements – expanding now 20 years. In this paper we present the physical activity patterns over these 20 years, as follow-up of Picavet et al. [9]. In addition, weight status and dietary behaviour are added as potential characteristics related to physical activity patterns.

In short, this study aims to describe individual leisure-time physical activity patterns of Dutch adults over a 20-year period, and to compare baseline socio-demographic, lifestyle, and health-related characteristics of participants with different patterns.

## Methods

### Study population

**Fig. 1:**
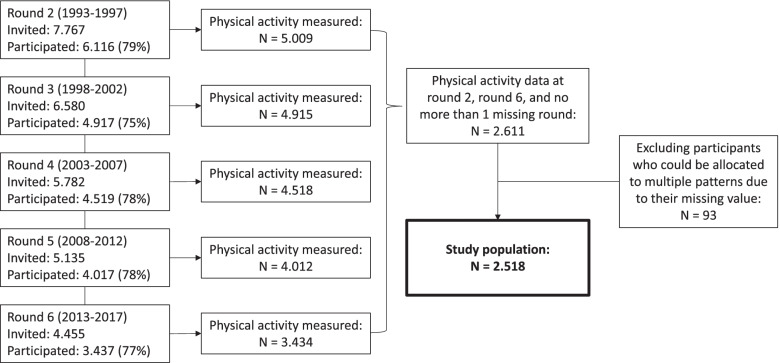
Flowchart of the measurement rounds of the Doetinchem Cohort Study

The Doetinchem Cohort Study (DCS) is an ongoing prospective observational study among a representative adult sample in the town of Doetinchem, in the eastern part of the Netherlands. Detailed information about the study can be found in the Cohort Profile [10] and its update [11]. The first measurement round took place between 1987 and 1991 and included 12,404 participants between 20 and 59 years old. For the second examination a random sample of 7,767 of the baseline participants (26–65 years old) were invited between 1993 and 1997. The second measurement round is regarded as the baseline measurement in this study, because the first assessment of physical activity took place in this round. Participants were measured once every five years, up until the sixth measurement round between 2013 and 2017, which included 3,437 participants aged 45–85 years old. A flowchart of the measurement rounds is shown in Fig. 1. Written informed consent was obtained from all participants. The DCS was approved by the Medical Ethics Committee of the University Medical Center Utrecht.

### Measurements

Measurements in the DCS consist of a questionnaire that participants complete themselves and an on-site physical examination by trained staff, including a review of the questionnaire.

### Physical activity

Leisure-time physical activity was assessed with a questionnaire designed for the European Prospective Investigation Into Cancer and Nutrition (EPIC), [12] which was extended with questions on sports and other strenuous physical activities. The questionnaire assesses time spent on walking, cycling, gardening and doing odd jobs in a regular week, for winter and summer separately. In addition, the questionnaire assesses time spent on a maximum of three sports or other strenuous activities in a regular week.

Based on the questionnaire data, participants were classified as ‘active’ or ‘inactive’ at each measurement round, based on the time they spent on moderate to vigorous intensity physical activities. This classification was in line with the previous study by Picavet and colleagues [9]. Activities included were cycling, gardening, and sports ≥ 4 metabolic equivalents. To account for the usual over-reporting of physical activities in questionnaire-based research, the smallest estimate per week for either summer or winter was used for cycling and gardening, and the cut-point for being classified as physically active was set at 210 min per week, instead of the recommended 150 min per week [9]. Sensitivity analyses were conducted with a cut-point of 150 min per week.

Subsequently, participants were allocated to one of five (predefined) physical activity patterns. Participants who were active in measurement round 2, at least twice in rounds 3–5, and in round 6, were classified as ‘Stable Active’. Participants who were inactive in measurement round 2, became active in round 3–5, and stayed active until round 6, were classified as ‘Becoming Active’. Participants who were active in measurement round 2, became inactive in round 3–5, and stayed inactive until round 6, were classified as ‘Becoming Inactive’. Participants who were inactive in measurement round 2, at least twice in rounds 3–5, and in round 6, were classified as ‘Stable Inactive’. All other participants were classified as ‘Varying.’ The classifications allowed for a maximum of one missing value in measurement rounds 3–5. Participants with missing physical activity values in measurement round 2 or 6, or with more than one missing physical activity value in rounds 3–5 were excluded. In addition, participants who could be allocated to multiple physical activity patterns due to their missing value were also excluded.

### Baseline characteristics

For all baseline characteristics, data from measurement round 2 (1993–1997) were used. Participants’ weight and height were measured during the physical examination and used to calculate BMI (kg/m^2^). Subsequently, BMI was classified into three categories of weight status: normal weight (including underweight; BMI < 25), moderately overweight (BMI ≥ 25 – < 30) or obese (BMI ≥ 30).

Dietary intake was assessed using the 178-item validated food frequency questionnaire (FFQ) from EPIC [13, 14]. The questionnaire was based on average consumption during the previous twelve months. Data from the FFQ were used to calculate the Dutch Healthy Diet index, [15] a measure of adherence to the Dutch Dietary Guidelines 2015 [16]. These guidelines include specific recommendations for 15 food groups, for example to consume ≥ 200 g of fruits per day. As the EPIC FFQ provided information on 13 out of the 15 food groups, the healthy diet index in the current study was based on 13 food groups. The score for each food group ranged from 0 to 10, so the index ranged from 0 (indicating no adherence to the guidelines) to 130 (indicating full adherence). The index was classified into quintiles.

Sex was based data from the registrations, which is sex assigned at birth. Age was divided into four 10-year categories at baseline: 26–35 years, 36–45 years, 46–55 years, 56–65 years. Level of education was classified as low (< intermediate secondary education equivalent to 6 years of education or less), intermediate (< intermediate vocational or higher secondary education), or high (higher vocational education/ university, equivalent to 16 years of education or more). Marital status was classified as married or not married (widowed/divorced/never been married). Work status was classified as employed (self-employed/salaried employment) or unemployed (unable to work/retired/homemaker/other). Smoking behaviour was classified as smoking or not smoking (including ex-smokers). Alcohol consumption was classified as drinking less than one glass per week or drinking one or more glass(es) per week. Self-perceived health was classified as good (excellent/very good/good) or moderate/poor.

### Statistical Analyses

Descriptive analyses were used to explore baseline characteristics and the proportion of participants belonging to each physical activity pattern. Multivariate logistic regression models were used to assess the associations between baseline socio-demographic, lifestyle, and health-related characteristics and the different physical activity patterns. In these analyses, the ‘Stable Active’ physical activity pattern was the reference for all other physical activity patterns. Data analyses were performed using Statistical Analysis System (SAS version 9.4). A two-sided p-value of < 0.05 was considered as statistically significant.

## Results

Physical activity was measured among 5009 participants in round 2 and 3434 in round 6 (see Fig. 1). Participants with valid physical activity values in measurement round 2 and 6, and not more than one missing physical activity value in measurement rounds 3–5 were included (*N* = 2,611). Participants that could not be allocated to one physical activity pattern due to their missing value (*N* = 93) were also excluded, leaving a sample of 2,518 participants. Baseline characteristics of the sample are shown in Table 1. The analytical sample included slightly more women (53%) than men and the mean (SD) age was 43.5 (9.0) years. Compared to the total group measured in round 2 the analytical sample is slightly younger, higher educated and healthier: less smoking, less overweight and higher perceived health as good. Among those in the analytical sample the percentage with physical activity is slightly higher (54.8%) than the total group in round 2 (52.0%).

**Table 1:** Baseline characteristics of the participants

**Characteristic**	Percentage or mean and standard deviation (SD)	
	Analytical sample	Round 2 (baseline)
**Population**	2,518	5,009
**Sex** *(% women)*	52.9%	53.2%
**Age** *(mean* ± *SD)*	43.5 ± 9.0	45.6 ± 10.0
**Age categories**		
26–35 years	20.2%	16.9%
36–45 years	37.6%	32.7%
46–55 years	30.8%	29.7%
56–65 years	11.6%	20.7%
**Level of education** *(%)*		
Low	46.4%	55.2%
Intermediate	30.3%	26.6%
High	23.3%	18.2%
**Marital status** *(%)*		
Married	82.7%	80.2%
**Work status** *(%)*		
Employed	73.6%	63.7
**Smoking** *(%)*	26.3%	31.8%
**Alcohol consumption** *(%)*		
≥ 1 glass per week	65.6%	62.1%
**Dutch Healthy Diet index** *(%)*^*a*^		
Quintile 1 (17–54)	20.0%	23.5%
Quintile 2 (54–61)	20.0%	19.1%
Quintile 3 (61–68)	20.0%	19.7%
Quintile 4 (68–75)	20.0%	19.1%
Quintile 5 (75–115)	20.0%	18.6%
**Weight status** *(%)*		
Normal weight (BMI < 25)	54.9%	49.0%
Moderately overweight (BMI ≥ 25 – < 30)	37.8%	40.0%
Obese (BMI ≥ 30)	7.4%	11%
**Perceived health** *(%)*		
Good	91.4%	87.6%
Moderate/poor	8.6%	12.5%
**Physical activity** *(%)*		
Active	54.8%	52.0%

^a^A higher score indicates better adherence to the Dutch Dietary Guidelines 2015

**Fig. 2:**
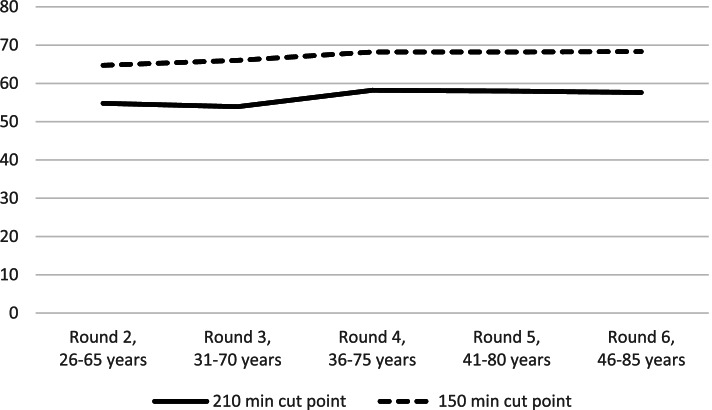
The percentage meeting the physical activity recommendations per measurement round

During the 20-year period, the overall percentage of participants that were classified as physically active was quite stable, with figures between 55 to 58% (Fig. 2) for the 210 min cut-off point. During this period, 33% of the participants were stable active, 20% were stable inactive, 15% were inactive at baseline but became active, 12% were active at baseline but became inactive, and 21% varied in their physical activity behaviour across time (Table 2). Using the 150 min cut-off point gives an higher stable activity-figure of 48.2% at expense of stable inactivity and the varying categories. The size of the ‘becoming active’ and ‘becoming inactive’-categories did hardly differ.

**Table 2:** Physical activity patterns of the participants

Pattern	Measurement in			Based on 210 min activity		Based on 150 min activity	
	**round 2**	**rounds 3—5**	**round 6**	*N*	% (95% CI)	*N*	% (95% Cl)
**Stable Active**	Active	Active in ≥ 2 rounds	Active	820	32.6 (30.8–34.4)	1213	48.2 (46.3–50.2)
**Becoming Active**	Inactive	Became and stayed active	Active	382	15.2 (13.8–16.6)	339	13.5 (12.2–14.8)
**Becoming Inactive**	Active	Became and stayed inactive	Inactive	292	11.6 (10.8–12.9)	288	11.4 (10.2–12.6)
**Stable Inactive**	Inactive	Inactive in ≥ 2 rounds	Inactive	500	19.9 (18.3–21.5)	278	11.0 (9.8–12.2)
**Varying**^**a**^	Active/inactive	Active/inactive	Active/inactive	524	20.8 (19.2–22.4)	401	15.9 (14.5–17.3)

^a^Participants were classified as ‘Varying’ if they did not fit any of the other physical activity patterns. *CI*  Confidence Interval

The results of the multivariate analyses of baseline socio-demographic, lifestyle and health-related characteristics and the different physical activity patterns are presented in Table 3. Compared to the ‘Stable Active’ pattern, participants who became active were more likely to be 46–55 years old, have an intermediate level of education, and to be smoking. Participants who became inactive were less often 46–55 years old and more often obese than participants with a ‘Stable Active’ pattern. The ‘Stable Inactive’ pattern was associated with an intermediate level of education, low adherence to the dietary guidelines, smoking, low levels of alcohol use and a moderate/poor perceived health. Participants in the ‘Varying’ physical activity pattern, finally, were more likely to have a low adherence to the dietary guidelines and to smoke than participants in the ‘Stable Active’ pattern.

**Table 3:** Multivariate odds ratio (95% confidence interval) of baseline socio-demographic, lifestyle and health-related characteristics with four physical activity patterns based on 210 min cut-off. Reference = ‘Stable Active’ (*N* = 820)

	**Becoming Active** (*N* = 382)	**Becoming Inactive** (*N* = 292)	**Stable Inactive** (*N* = 500)	**Varying Physical Activity** (*N* = 524)
**Sex**				
Women	1.00	1.00	1.00	1.00
Men	1.08 (0.77–1.53)	0.77 (0.53–1.13)	1.10 (0.80–1.52)	0.77 (0.56–1.05)
**Age**				
26–35	1.00	1.00	1.00	1.00
36–45	1.13 (0.72–1.79)	0.68 (0.43–1.08)	0.79 (0.53–1.17)	0.81 (0.56–1.19)
46–55	**1.71 (1.06–2.75)**	**0.48 (0.29–0.81)**	0.79 (0.51–1.22)	0.76 (0.50–1.15)
56–65	1.86 (0.94–3.68)	0.83 (0.41–1.66)	1.35 (0.75–2.45)	0.92 (0.51–1.66)
**Level of education**				
Low	1.00	1.00	1.00	1.00
Intermediate	**1.69 (1.17–2.44)**	0.89 (0.59–1.36)	**1.46 (1.03–2.06)**	1.32 (0.94–1.84)
High	1.06 (0.72–1.57)	0.70 (0.45–1.10)	1.03 (0.71–1.50)	1.12 (0.79–1.59)
**Marital status**				
Not married	1.00	1.00	1.00	1.00
Married	1.25 (0.81–1.93)	1.37 (0.85–2.20)	1.01 (0.69–1.47)	0.88 (0.62–1.25)
**Work status**				
Unemployed	1.00	1.00	1.00	1.00
Employed	1.28 (0.84–1.97)	0.76 (0.49–1.16)	0.97 (0.65–1.43)	0.94 (0.66–1.34)
**Smoking behaviour**				
Not smoking	1.00	1.00	1.00	1.00
Smoking	**1.44 (1.01–2.06)**	1.09 (0.73–1.64)	**1.42 (1.02–2.00)**	**1.43 (1.04–1.97)**
**Alcohol consumption**				
< 1 glass p/week	1.00	1.00	1.00	1.00
≥ 1 glass p/ week	0.75 (0.54–1.06)	1.15 (0.78–1.68)	**0.62 (0.46–0.85)**	0.88 (0.65–1.19)
**Dutch Healthy Diet**				
**Index**^**a**^				
Quintile 1 (17–55)	1.67 (1.00–2.79)	1.62 (0.94–2.80)	**2.33 (1.44–3.74)**	**1.62 (1.02–2.57)**
Quintile 2 (55–61)	1.34 (0.82–2.17)	0.97 (0.57–1.66)	1.24 (0.79–1.97)	1.34 (0.88–2.05)
Quintile 3 (61–68)	0.77 (0.47–1.27)	0.99 (0.59–1.65)	0.97 (0.62–1.52)	1.05 (0.69–1.60)
Quintile 4 (68–75)	1.12 (0.70–1.78)	0.72 (0.42–1.23)	0.83 (0.52–1.32)	0.98 (0.64–1.49)
Quintile 5 (75–115)	1.00	1.00	1.00	1.00
**Weight status**				
Normal weight (BMI < 25)	1.00	1.00	1.00	1.00
Moderately overweight	0.77 (0.55–1.08)	1.08 (0.75–1.56)	0.95 (0.69–1.30)	1.28 (0.96–1.71)
(BMI ≥ 25 – < 30)				
Obese (BMI ≥ 30)	1.55 (0.86–2.78)	**1.90 (1.01–3.57)**	1.63 (0.93–2.85)	1.42 (0.81–2.48)
**Perceived health**				
Good	1.00	1.00	1.00	1.00
Moderate/poor	0.85 (0.43–1.67)	1.73 (0.90–3.31)	**2.51 (1.52–4.16)**	1.53 (0.90–2.60)

^a^A higher score indicates better adherence to the Dutch Dietary Guidelines 2015

Statistically significant results (*p* < 0.05) are highlighted in bold

The sensitivity analyses (results not presented) with a cut-point of 150 min of physical activity per week (instead of 210 min per week) showed an increase of around 10% in the proportion of participants meeting the physical activity guidelines in all measurement rounds. Using this cut-point, more participants were classified as ‘Stable Active’ while less participants were classified as ‘Stable Inactive’ or ‘Varying.’ The results of the multivariate analyses were reasonably similar.

## Discussion

This study aimed to describe individual leisure-time physical activity patterns among adults over a 20-year period, and to compare baseline characteristics of participants with different patterns. Over a 20-year period, 33% of the study population were stable active, 20% were stable inactive, 15% became active, 12% became inactive, and 21% had varying physical activity levels. Becoming active was associated with being 46–55 years old, having an intermediate level of education, and being a current smoker. Participants who became inactive were less likely to be 46–55 years old and more likely to be obese. Stable inactivity was associated with an intermediate level of education, low adherence to the dietary guidelines, smoking, low levels of alcohol use and a moderate/poor perceived health. Participants with a varying physical activity level were more likely to have low adherence to the dietary guidelines and to smoke.

The current study adds 10 years to the data presented by Picavet and colleagues [9]. Their prevalence numbers for stable activity (31%), stable inactivity (24%), becoming active (18%), becoming inactive (15%) and varying physical activity (13%) are reasonably comparable to the results of the current analyses. The most distinct difference is the larger proportion of participants with a varying physical activity level in the current study, which can probably be contributed to the larger follow-up period with more measurement points, which increases the chance of varying physical activity patterns.

The observation that most participants (53%) showed stable physical activity patterns is in line with the results of a recent systematic literature review by Lounassalo and colleagues [17]. They included 27 longitudinal studies on trajectories of physical activity and reported that, in adults, stable physical activity trajectories (both active and inactive) were more prevalent than changing trajectories. However, almost half of the current study population did change their physical activity over a 20-year period, indicating that physical activity is a dynamic behaviour that can change throughout the life course.

The current study showed associations between baseline socio-demographic, lifestyle and health-related characteristics and different physical activity patterns. Compared to participants with a stable physical activity pattern, participants who were stable inactive were more likely to have an intermediate level of education, current smoking behaviour, low alcohol consumption, low adherence to dietary guidelines, and moderate/poor perceived health. In general, these findings are in line with the systematic literature review by Lounassalo and colleagues who also investigated factors related to trajectories of physical activity, [17] and a systematic literature review by Trost and colleagues about correlates of adults’ participation in physical activity [18]. Both reviews, however, identified sex as an important factor, reporting that men are more physically active than women, while the current study did not find a sex difference for any of the physical activity patterns, nor in baseline physical activity behaviour. While this contrasts previous international research, it is in line with trend data in the Netherlands, which show comparable percentages of meeting the physical activity guidelines for men and women [8].

In the current study, approximately one-third of the population was either stable inactive or became inactive in the 20-year period and could therefore benefit from strategies aiming to increase (or sustain) their physical activity. Stimulating physical activity in later life is worthwhile, as studies have shown that increasing leisure-time physical activity during the life course might positively effect mortality levels [19, 20]. In addition, the current study showed that 15% of the participants became active, which shows that it is possible to change physical activity habits in adulthood. Future research should therefore not only focus on effective strategies to increase but also to sustain physical activity levels in (older) adults.

### Strengths and limitations

The main strength of this study is the use of longitudinal physical activity data, that was collected in multiple successive measurement rounds in a similar manner over 20 years. This provided the opportunity to study individual changes in physical activity levels over an extended follow-up period.

The main limitation of this study is the fact that physical activity was assessed using a self-report measure. It is well known that self-reported physical activity can suffer from recall bias and social-desirability bias, which usually leads to an overestimation of physical activity. However, to account for the over-reporting of physical activity, the smallest estimate for either summer or winter was used for cycling and gardening, and the overall cut-point was set at 210, instead of 150, minutes per week, the same as in the earlier study based on the first three rounds [9]. In addition, as the same questionnaire was used in every measurement round, the physical activity estimates are expected to be comparable across rounds. However, the bias may differ per measurement round because individuals are ‘trained’ to respond to the questionnaire every 5 years, and also the notion of relevance of physical activity has changed which may also affect the (social desirability) in response to the questionnaire. It is not known how this possible change in perspective has affected our findings. It will be very interesting to see the results of future research using device-based measures of physical activity to assess longitudinal changes of individual physical activity patterns, which might be less affected by reporting bias.

Another limitation refers to selection bias because those participating in long-term health studies are relatively more often healthier and higher educated. This was also true for this study [11]. This implies that the figures for the general population are unhealthier than estimated in this sample, with at least meaning that the proportion of those that are stable active is probably lower.

Finally, the range of baseline characteristics included in the current analyses was limited. Future research should therefore study additional characteristics that are known to be associated with physical activity behaviour, such as genetic factors, sleep, and environmental factors, in relation to individual physical activity patterns (Lounassalo 2021). Moreover, in order to inform strategies aiming to increase—or sustain—physical activity, future studies should explore why people are—or become—physically inactive. For this purpose, a combination of quantitative and qualitative research is recommended.

## Conclusions

Over a period of 20 years, approximately one-third of the adult study population was stable physically active, one-fifth was stable inactive, while almost half of the population changed their physical activity level. This indicates that physical activity is a dynamic behaviour that can change throughout the life course. Baseline age, level of education, smoking, alcohol consumption, adherence to dietary guidelines, weight status and perceived health were associated with different physical activity patterns. Future research should be focused on strategies aiming to increase, or sustain, physical activity levels of adults.

## Data Availability

The data that support the findings of this study are available from the Doetinchem Cohort Study but restrictions apply to the availability of these data, which were used under license for the current study, and so are not publicly available. Data are however available from the authors upon reasonable request and with permission of the Doetinchem Cohort Study.

## References

[1] World Health Organization. Global recommendations on physical activity for health. Geneva: World Health Organisation; 2010. 26180873

[2] World Health Organization. Global Health Risks: Mortality and burden of disease attributable to selected major risks. 2009.

[3] Lee IM, Shiroma EJ, Lobelo F, Puska P, Blair SN, Katzmarzyk PT. Effect of physical inactivity on major non-communicable diseases worldwide: an analysis of burden of disease and life expectancy. Lancet. 2012;380(9838):219–29. 10.1016/S0140-6736(12)61031-9 PMC3645500 22818936

[4] Bull FC, Al-Ansari SS, Biddle S, Borodulin K, Buman MP, Cardon G (2020). World Health Organization 2020 guidelines on physical activity and sedentary behaviour. Br J Sports Med.

[5] Hallal PC, Andersen LB, Bull FC, Guthold R, Haskell W, Ekelund U (2012). Global physical activity levels: surveillance progress, pitfalls, and prospects. The Lancet.

[6] Guthold R, Stevens GA, Riley LM, Bull FC (2018). Worldwide trends in insufficient physical activity from 2001 to 2016: a pooled analysis of 358 population-based surveys with 1·9 million participants. Lancet Glob Health.

[7] Ding D, Lawson KD, Kolbe-Alexander TL, Finkelstein EA, Katzmarzyk PT, van Mechelen W (2016). The economic burden of physical inactivity: a global analysis of major non-communicable diseases. The Lancet.

[8] Dutch website with the monitoring figures on physical activity in the Netherlands [https://www.sportenbewegenincijfers.nl/kernindicatoren/beweegrichtlijnen]. Accessed 31 May 2021.

[9] Picavet HSJ, Wendel-Vos GCW, Vreeken HL, Schuit AJ, Verschuren WMM (2011). How Stable Are Physical Activity Habits among Adults? The Doetinchem Cohort Study. Med Sci Sports Exerc.

[10] Verschuren WMM, Blokstra A, Picavet HSJ, Smit HA (2008). Cohort profile: the Doetinchem Cohort Study. Int J Epidemiol.

[11] Picavet HSJ, Blokstra A, Spijkerman AMW, Verschuren WMM (2017). Cohort Profile Update The Doetinchem Cohort Study 1987–2017 lifestyle, health and chronic diseases in a life course and ageing perspective. Int J Epidemiol.

[12] Pols MA, Peeters PHM, Ocké MC, Slimani N, Bueno-De-Mesquita HB, Collette HJA (1997). Estimation of Reproducibility and Relative Validity of the Questions Included in the EPIC Physical Activity Questionnaire. Int J Epidemiol.

[13] Ocké MC, Bueno-De-Mesquita HB, Goddijn HE, Jansen A, Pols MA, van Staveren WA (1997). The Dutch EPIC Food Frequency Questionnaire I Description of the Questionnaire, and Relative Validity and Reproducibility for Food Groups. Int J Epidemiol.

[14] Ocké MC, Bueno-De-Mesquita HB, Pols MA, Smit HA, van Staveren WA, Kromhout D (1997). The Dutch EPIC Food Frequency Questionnaire II Relative Validity and Reproducibility for Nutrients. Int J Epidemiol.

[15] Looman M, Feskens EJ, de Rijk M, Meijboom S, Biesbroek S, Temme EH (2017). Development and evaluation of the Dutch Healthy Diet index 2015. Public Health Nutr.

[16] Kromhout D, Spaaij CJ, de Goede J, Weggemans RM (2016). The 2015 Dutch food-based dietary guidelines. Eur J Clin Nutr.

[17] Lounassalo I, Salin K, Kankaanpaa A, Hirvensalo M, Palomaki S, Tolvanen A (2019). Distinct trajectories of physical activity and related factors during the life course in the general population: a systematic review. BMC Public Health.

[18] Trost SG, Owen N, Bauman AE, Sallis JF, Brown W (2002). Correlates of adults’ participation in physical activity: review and update. Med Sci Sports Exerc.

[19] Byberg L, Melhus H, Gedeborg R, Sundstrom J, Ahlbom A, Zethelius B (2009). Total mortality after changes in leisure time physical activity in 50 year old men 35 year follow-up of population based cohort. BMJ.

[20] Talbot LA, Morrell CH, Fleg JL, Metter EJ (2007). Changes in leisure time physical activity and risk of all-cause mortality in men and women: the Baltimore Longitudinal Study of Aging. Prev Med.

